# Dr. Benjamin Howard’s Direct Method of Artificial Respiration

**Published:** 1869-08

**Authors:** 


### Dr. Benjamin Howard's Direct Method of Artificial Respiration.

"The patient is laid on the ground upon his back, his arms fully extended backward and outward, a firm roll of clothing being placed beneath the false ribs, so as to throw their anterior margin prominently forward. The tongue being held forward by an assistant the operator facing the patient, kneels astride his abdomen, and places both hands so as the balls of the thumbs rest on the anterior margins of the false ribs, the four fingers falling naturally into four of the lower corresponding intercostal spaces on each side.

"The elbows of the operator being then planted against his sides, he has but to throw himself forward, using his knees as a pivot, and the entire weight of his trunk is brought to bear upon the patient's false ribs. If, at the same time, the fingers of the operator grasp and squeeze the false ribs toward each other, these combined actions crowd the false ribs upward and inward, producing the greatest possible motion of the diaphragm and displacement of the contents of the pulmonary air-cells.

"The operator then suddenly lets go and returns upon his knees, where both the in-rush of air and the natural elasticity of the ribs at this part cause instant return to their normal position.

"This repeated with proper rhythm and frequency, constitutes the entire process."—*Medical Archives.*

